# Healthy and Safe Swimming Week — May 22–28, 2017

**DOI:** 10.15585/mmwr.mm6619a1

**Published:** 2017-05-19

Healthy and Safe Swimming Week highlights measures that swimmers, parents of young swimmers, aquatic facility (e.g., swimming pool and support infrastructure) operators, residential pool or hot tub/spa owners, beach managers, and public health officials can take to maximize the health benefits of water-based physical activity while minimizing the risk for recreational water–associated illness and injury. A public health communications toolkit is available at https://www.cdc.gov/healthywater/observances/hss-week/response-tools-public-health.html.

The theme of this year’s observance is Diarrhea and Swimming Don’t Mix. C*ryptosporidium*, a parasite that causes profuse, watery diarrhea, has emerged as the leading etiology of recreational water–associated outbreaks, particularly those associated with aquatic facilities (*1*). This issue of *MMWR* includes a report on *Cryptosporidium* molecular characterization, highlighting its utility in investigating these outbreaks (*2*).

In July 2016, CDC released the 2016 Model Aquatic Health Code (MAHC) (https://www.cdc.gov/mahc/editions/current.html). This national guidance can be voluntarily adopted by state and local jurisdictions to minimize the risk for public aquatic facility–associated illness and injury. The MAHC guidance reflects biennial input from public health professionals and other stakeholders through the Council for the MAHC (https://www.cmahc.org).
